# Evaluation of Knowledge, Attitude, and Practice of Iranian Medical Students Toward Complementary and Alternative Medicine: A Cross‐Sectional Survey

**DOI:** 10.1002/hsr2.70539

**Published:** 2025-03-12

**Authors:** Salman Vojdani, Seyede Maryam Najibi, Binazir Niknam, Babak Daneshfard, Mohammad Salehi‐Marzijarani, Ramin Nasimi Doost Azgomi, Mohammad Hashem Hashempur

**Affiliations:** ^1^ Department of Anesthesiology and Pain Medicine Fasa University of Medical Sciences Fasa Iran; ^2^ Research Center for Traditional Medicine and History of Medicine, Department of Persian Medicine, School of Medicine Shiraz University of Medical Sciences Shiraz Iran; ^3^ Student Research Committee Shiraz University of Medical Sciences Shiraz Iran; ^4^ Chronic Respiratory Diseases Research Center, National Research Institute of Tuberculosis and Lung Diseases (NRITLD) Shahid Beheshti University of Medical Sciences Tehran Iran; ^5^ Canadian College of Integrative Medicine (CCIM) Montreal Quebec Canada; ^6^ Persian Medicine Network (PMN) Universal Scientific Education and Research Network (USERN) Tehran Iran; ^7^ Cancer Epidemiology Research Center AJA University of Medical Sciences Tehran Iran; ^8^ Clinical Trial Center AJA University of Medical Sciences Tehran Iran; ^9^ Traditional Medicine and Hydrotherapy Research Center Ardabil University of Medical Sciences Ardabil Iran

**Keywords:** attitude, complementary and alternative medicine, integrative medicine, Iran, knowledge, practice, traditional Persian medicine

## Abstract

**Background and Aims:**

Complementary and alternative medicine (CAM) encompasses a wide variety of health practices and products that are generally not included in conventional medicine. This study evaluates knowledge, attitude, and practice (KAP) among medical students toward CAM in Iran.

**Methods:**

In this cross‐sectional study, we developed and used a questionnaire to evaluate the KAP of medical students regarding CAM. The sampling method was convenience sampling. Two hundred and twenty medical students who were in their first to three (junior) and the last 2 years of education (senior) participated in this study. The data was collected from January to May 2017. Data analysis employed descriptive statistics, chi‐square test, and independent *t*‐test. Statistical significance was set at *p*‐values less than 0.05.

**Results:**

Of 220 medical students, 125 respondents were CAM users (56.81%). The score of attitude about CAM was 60.13 ± 10.48 in junior students and 60.81 ± 7.32 among senior students. The score of knowledge about CAM was significantly (*p* = 0.02) higher in senior students (9.05 ± 4.12) than in juniors (7.74 ± 5.23). Traditional Persian medicine (TPM) was found to be the most commonly practiced CAM by both groups of students and their families. Leeching in students and chiropractic in their families had the lowest CAM use rates.

**Conclusion:**

Although CAM knowledge was relatively low in medical students, they have a positive attitude toward it. TPM was the most common practice among CAM approaches. It is necessary to incorporate CAM modalities especially TPM education into the curriculum of medical students and other healthcare disciplines to enhance students' knowledge and attitude toward CAM and its role in healthcare.

## 1 Introduction

Complementary and alternative medicine (CAM) comprises a wide range of treatment modalities including drugs and other therapeutic products which at the current time are not accounted as a part of conventional medicine (CM) [1, 2]. CAM is categorized into five main groups including mind‐body therapies, energy therapy, biologically based treatments, bodily manipulations, and alternative medical systems [3]. These practices are significantly influenced by the culture and traditions of various communities and play an important role in healthcare systems worldwide [4, 5, 6].

International studies and some reports from the World Health Organization (WHO) are confirming the improving interests of people in different nations regarding CAM modalities [7, 8]. The use of CAM differs significantly across countries. A systematic review in 14 different countries found substantial CAM use, ranging from 24% to 71.3% across various nations [9]. The studies among patients with thyroid diseases and diabetes found that 85.4% and 51% reported using CAM, respectively [10, 11]. CAM use is influenced by a variety of factors, both at the individual level and at the national level. Socioeconomic status, demographic characteristics, being a woman, health indicators, higher educational level and health expenditures all significantly affect the utilization of CAM [12, 13]. Also, a recent systematic review conducted worldwide found that certain populations, such as cancer patients and individuals with diabetes, are more likely to use CAM compared to the general population [14]. Moreover, patients with chronic diseases utilize CAM more frequently than those without such conditions [15, 16, 17, 18]. According to the studies, the main reasons for CAM use are expecting benefits of CAM, dissatisfaction with CM, and the perceived safety of CAM [14, 19].

Although patients are generally satisfied with and accept CAM, its specific position remains unclear. It is necessary to improve knowledge and attitude at both university and community level [20]. The beliefs and attitudes of medical students as future physicians toward CAM are significant and may influence their inclination to engage in CAM services actively. Therefore, understanding the level of knowledge, attitude, and practice (KAP) of medical students regarding CAM would be useful for educational policymakers in reorganizing the curricular concepts and improving the training and their level of knowledge [21, 22].

KAP studies are considered valuable methods for the evaluation of healthcare delivery across various fields [23]. Numerous studies have assessed medical students' KAP regarding CAM. Such a survey conducted in Canada revealed that 90% of the medical student will discuss CAM with their patients; while only 40% believed they had sufficient knowledge of the subject [24]. Similarly, a study in Germany found that 40% of students felt they needed more information on CAM modalities and advocated for their inclusion in the educational curriculum [25]. Additionally, 31% of medical students in Iran were utilizing CAM modalities, and half of them expressed a desire for training in these treatments [26].

With the rising popularity of CAM among patients, medical students must be knowledgeable about these practices to ensure comprehensive patient care. Minimal understanding of CAM modalities can hinder their ability to refer patients appropriately and engage in informed discussions about CAM options [27]. Assessing medical students' KAP helps in determining the level of awareness and acceptance of CAM therapies among future healthcare professionals. Also, it allows for the identification of gaps in education and training related to CAM [28]. Furthermore, studying medical students' KAP toward CAM can provide valuable insights into their willingness to integrate CAM into their future medical practice, which could ultimately influence patient care and treatment choices [29]. Therefore, this study evaluates medical students' KAP on CAM at Fasa University of Medical Sciences.

## 2 Methods

### 2.1 Study Design

This research was a cross sectional‐analytical study conducted on medical students at Fasa University of Medical Sciences. In the first stage, we created a questionnaire to evaluate the KAP of medical students regarding CAM. In the next step, we assessed their KAP regarding CAM by using the developed questionnaire.

### 2.2 Data Collection

To design the questionnaire we used a literature review to develop a list of potential questions. Then, duplicate and unnecessary questions were removed from the initial list. Nine experts were invited to assess the content validity of the provided initial list of questions. The inclusion criteria for experts included practitioners of traditional Persian medicine (TPM), renowned researchers in CAM, distinguished methodology researchers, and willingness to participate in the study. They assessed each question for necessity, relatedness, clarity, and simplicity to calculate the content validity ratio (CVR) and content validity index (CVI). The scores for CVR and CVI were 0.79 and 0.93, respectively, indicating good content validity. We also tested the internal consistency of the questionnaire using Cronbach's *α*, which showed the value of 0.82. Data were collected using a self‐report questionnaire (see Supporting Information S1: Additional File 1).

### 2.3 Measures

The final version of the questionnaire has 40 questions and consists of four parts: demographics, CAM attitude, use or practice, and medical students' knowledge.

#### 2.3.1 Demographic Section

This section includes questions about age, gender, years of education, and marital status.

#### 2.3.2 CAM Attitude

This part comprises 24 questions, and each question was scored using a five‐point Likert scale (strongly agree, agree, neither agree nor disagree, disagree, and strongly disagree) and assessed the individual's attitude toward CAM modalities. Out of the 24 questions, six questions assessed negative attitudes and thus were scored in reverse. The lowest total score and the highest total score were 24 and 72, respectively.

#### 2.3.3 CAM Use or Practice

This section checks whether participants used the eight commonly used CAM modalities in Iran (TPM, acupuncture, homeopathy, Yoga, cupping, leech therapy, massage therapy, and chiropractic).

#### 2.3.4 CAM Knowledge

This part assesses the participants' level of knowledge about 8 commonly used CAM modalities in Iran (TPM, acupuncture, homeopathy, Yoga, cupping, leech therapy, massage therapy, chiropractic). The scoring scale of this section was a 5‐point Likert scale (very familiar to completely unfamiliar) with a question: please indicate your familiarity level with the following items using the available options. In this section, the lowest total score and the highest total score were 0 and 32, respectively.

### 2.4 Sampling and Statistical Analysis

We used a non‐probabilistic sampling method, that is, convenience sampling. The inclusion criteria were being a medical student at Fasa University of Medical Sciences in first, second, third years (junior), sixth, and seventh year (senior) of education and willingness to participate in the study. Participants who did not fully complete their questionnaires and guest students were excluded from the study. Years of education were considered because a medical student's knowledge and attitudes may change throughout their training. The KAP scores were calculated and converted into percentages. Bloom's cutoff points were used to categorize KAP levels as good/positive (≥ 80%), moderate/neutral (60%–79%), and poor/negative (≤ 59%) [30]. A total of 220 medical students participated in the survey. Quantitative and qualitative data were described by mean ± standard deviation (SD) and frequency (percent), respectively. The Chi‐square test and Mann−Whitney test were used for comparing qualitative and quantitative variables between the groups, respectively. The Statistical Package for the Social Sciences software version 15 (SPSS Inc., Chicago, IL, USA) was used for the analysis. All tests were two‐tailed and statistical significance was set at *p*‐values less than 0.05. We used the recommendations provided in Assel et al. to guide on the appropriate analysis, reporting, and interpretation of clinical research [31].

### 2.5 Ethical Considerations

This research received approval from the Local Medical Ethics Committee of Fasa University of Medical Sciences (ID: 94151), ensuring that all study procedures conformed to ethical standards for human research. To respect participants' autonomy and rights, a written informed consent was added to the questionnaires to reassure the participants regarding their privacy and dignity, in addition to a verbal explanation about the research project's goals and flow. Participants were assured that their personal information would not be disclosed. This commitment to ethical considerations aligns with the principles set forth in the Declaration of Helsinki.

## 3 Results

Of the total number of 220 medical students, 37.5% were in the first, 18.05% in the second, 18.05% in the third, 12.03% in the sixth, and 14.35% in the seventh year of medical education. Most of the participants were female (60%), single (85.32%) and 125 of medical students were CAM users (56.81%). The average age of the participants was approximately 21. Using the CAM was more prevalent among those students that their families were CAM users. Gender, age, marital status, and entrance year of university were not significantly associated with the use of CAM. However, CAM usage in family was significantly associated with the CAM use (*p* = 0.017). Table 1 represents the demographic characteristics of CAM users.

**Table 1:** Demographic data of participants based on their CAM usage.

Variable	CAM usage		*p* value^a^
	Yes (*n* = 125)	No (*n* = 95)	
	*N* (%)	*N* (%)	
Gender			0.19
Female	79 (63.2)	52 (54.7)	
Male	46 (36.8)	43 (45.2)	
Marital status			0.68
Married	17 (13.6)	15 (15.7)	
Single	108 (86.4)	80 (84.21)	
Age	21.91 ± 2.85	21.67 ± 2.7	0.55
CAM usage in family			0.017
Yes	106 (84.8)	68 (71.5)	
No	19 (15.2)	27 (28.4)	
Entrance year			0.49
1st	43 (34.4)	38 (40.0)	
2nd	23 (18.4)	16 (16.8)	
3rd	19 (15.2)	20 (21.0)	
6th	18 (14.4)	8 (8.4)	
7th	22 (17.6)	13 (13.6)	

^a^ Chi‐square and Mann−Whitney tests were used to compare qualitative and quantitative variables between groups, respectively.

The score of attitude about CAM was not significantly different (Cohen's *d* = 0.075, *p* = 0.60) between junior students (60.13 ± 10.48) and senior students (60.81 ± 7.32). However, the score of knowledge about CAM was significantly (Cohen's *d* = 0.278, *p* = 0.025) higher in senior students (9.05 ± 4.12) than juniors (7.74 ± 5.23) (Table 2).

**Table 2:** Knowledge and attitude of participants based on CAM usage.

	Medical students		Effect size^a^	*p* value^a^
	Junior students	Senior students		
Attitude score	60.13 ± 10.48	60.81 ± 7.32	0.075	0.60
Knowledge score	7.74 ± 5.23	9.05 ± 4.12	0.278	0.025

^a^ The Mann−Whitney test is used for comparing the scores between the groups. Effect sizes are calculated based on the Cohen's *d*.

The results indicated that, based on Bloom's cut‐off, 65.9% of the 220 medical students surveyed demonstrated good/positive attitude, while 96.8% displayed poor/negative knowledge levels. Additionally, among the respondents, 51.6% reported using at least 1−2 item CAM practices (Table 3).

**Table 3:** Categorized scores of knowledge, attitude, and practice in total medical students.

Total medical students			
	Categories	Number	Percent
Attitude	Poor/negative	6	2.7
	Moderate/neutral	69	31.4
	Good/positive	145	65.9
Knowledge	Poor/negative	212	96.8
	Moderate/neutral	7	3.2
	Good/positive	0	0
Practice	No CAM use	95	43.4
	1−2 item CAM use	113	51.6
	3−5 item CAM use	10	4.6
	6−8 item CAM use	1	0.5

**Figure 1:**
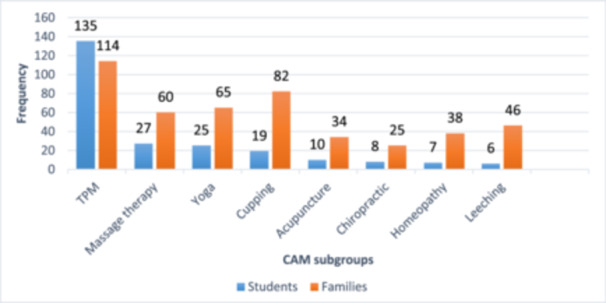
CAM subgroups used by students and their families.

Figure 1 depicts the different types of CAM used by students and their families. TPM was found to be the most commonly used by both students and families.

## 4 Discussion

The present study was conducted to evaluate Iranian medical students' KAP about CAM. The results revealed 125 of them were CAM users (56.81%). The use of CAM among medical students is a topic of interest in various countries and is not limited to a specific region or country. According to the report published by WHO the prevalence of CAM usage in 2018 was 89% in Europe, 80% in the Americas, and 95% in the Western Pacific region [7]. A systematic review found that the prevalence of CAM usage in medical specialities is 45% [32]. However, reported rates of CAM usage can vary significantly, which may be attributed to differing methods used to assess it [33]. One study identified several reasons for the use of CAM, including positive attitudes toward CAM, negative attitudes toward CM, and other influencing factors such as social networks, recommendations from physicians, having an internal health locus of control, and traditional beliefs [14]. However, it's important to note that CAM usage and the reasons behind it can differ based on the socio‐cultural values, geographic location, and family or community structures of different populations [34].

Based on the results, the score of knowledge about CAM was 7.74 for junior students and 9.05 for senior students. Also, 96.8% of medical students surveyed demonstrated poor/negative knowledge levels. It appears that medical students in Iran have poor knowledge about CAM. A systematic review found that nursing students had limited knowledge and understanding of CAM [35]. Similarly, a study conducted in Bangladesh represented that students had inadequate knowledge of CAM [28]. Generally, the limited understanding of CAM among students is often attributed to insufficient support for CAM education. This lack of knowledge is particularly concerning given the high prevalence of CAM use among the global population, including patients in Iran. Evidence suggests that this knowledge gap could hinder effective communication between future physicians and their patients regarding the use of CAM [36, 37]. Having a proper knowledge of CAM is essential for preventing potential drug and herbal interactions and providing better guidance to patients in the future [38].

In our study, the score of knowledge about CAM was significantly higher in senior students than in juniors. It may be that senior students typically have more exposure to CAM concepts through their coursework, clinical practice, and interactions with patients. This extended exposure allows them to accumulate more knowledge and practical understanding of CAM modalities compared to juniors who are still in the early stages of their education [39, 40]. Senior students may develop a greater personal interest in CAM, leading them to seek additional resources, attend workshops, or engage in self‐directed learning [41, 42].

In this study, the mean attitude score for CAM was approximately 60.38. Also, 65.9% of the 220 medical students surveyed displayed a good/positive attitude toward CAM. Similarly, Studies suggested that students from Libya [38], Iran [43], Turkey [44], and Indonesia [45] exhibited positive attitudes toward CAM usage. This is also noted among the university's academic staff members, as a study conducted in India found that medical faculty members also have different knowledge and attitudes. Many have reservations about recommending specific types of CAM therapies or integrating them with CM because there are barriers to CAM practice that need to be addressed [46]. Research indicates that educational exposure to CAM significantly influences students' attitudes toward it. Therefore, if students have limited opportunities to learn about CAM outside of the formal curriculum, it is crucial to provide them with adequate educational exposure [47, 48]. Incorporating formal courses on CAM into the curriculum can help the community understand the proper and improper use of CAM. This highlights the need to update academic curricula and health policies to regulate and standardize CAM healthcare practices for the safety of the public [49].

According to the results, the attitude score about CAM was not significantly different between junior and senior students. Studies indicate that attitudes may remain static across the education period, resulting in no significant difference. Cultural background and social influences might play a role in shaping attitudes toward CAM for both groups. If the broader cultural context promotes a balanced view of conventional and alternative medicine, this could lead to consistent attitudes across different academic years [47, 50].

The findings showed that TPM was the most commonly used type of CAM among both students and their families. Traditional and complementary medicine is deeply ingrained in Iranian culture, which means that doctors cannot ignore it so they need to have knowledge and a positive attitude toward it. This is particularly important for medical students and healthcare professionals, who need to manage their patients' conditions effectively. cultural differences can influence the usage type of CAM, which often reflects regional, socioeconomic, and educational variations; for example, the high use of yoga in Indian medical students [51], TCM in Chinese medical students [52], and TPM in Iranian medical students [26].

Notably, 65.9% of the 220 participants exhibited a good or positive attitude toward CAM, suggesting a prevailing openness among students to alternative approaches in healthcare. In stark contrast, a staggering 96.8% demonstrated poor or negative knowledge levels concerning CAM, highlighting a critical gap in educational content that may hinder informed decision‐making. Furthermore, despite this knowledge deficiency, 51.6% of respondents reported utilizing at least 1−2 items from CAM practices, indicating that many students engage with these modalities regardless of their understanding. This disconnect between favorable attitudes and inadequate knowledge underscores the need for enhanced educational programs within medical curricula to better equip students with the essential knowledge and critical appraisal skills necessary to navigate the complexities of CAM.

### 4.1 Strengths and Limitations of the Study

This study offers valuable insights into medical students' KAP regarding CAM and is the first to assess KAP among medical students in southern Iran. By utilizing a well‐constructed questionnaire, the research effectively captures the KAP landscape across various year groups. The meaningful sample size, which includes both junior and senior students, allows for a comprehensive analysis of trends across different educational levels. However, our research does have some limitations. This was a cross‐sectional study, so it is important to be cautious when generalizing the results. Also, CAM use, attitudes, and knowledge may be different and heterogeneous in various countries that makes generalizations difficult. However, important contextual differences in medical education programs and different groups of medical students across the globe contribute to the diversity of reported outcomes. Furthermore, this research was conducted with a small sample size relatively, longitudinal studies with larger sample sizes in other research and education settings are recommended.

## 5 Conclusion

In conclusion, while knowledge about CAM among Iranian medical students is relatively low, their attitudes toward CAM are notably positive. The attitude scores were 60.13 for junior students and 60.81 for seniors, with senior students demonstrating a higher level of knowledge compared to their junior counterparts. Factors such as gender, age, marital status, and entrance year to university were not significantly associated with CAM usage; however, family use of CAM was significantly linked to students' own CAM practices. TPM emerged as the most commonly utilized CAM by both student groups and their families, while leech therapy and chiropractic care ranked as the least used. This highlights an opportunity for policymakers to integrate popular CAM topics, especially TPM, into clinical education programs. Such integration will enhance students' capabilities in counseling and decision‐making as they encounter patients seeking CAM options.

## Author Contributions

**Salman Vojdani:** investigation, resources, supervision, writing – review and editing. **Seyede Maryam Najibi:** validation, formal analysis, writing – original draft. **Binazir Niknam:** investigation, writing – review and editing. **Babak Daneshfard:** methodology, writing – original draft. **Mohammad Salehi‐Marzijarani:** methodology, validation, formal analysis, writing – review and editing. **Ramin Nasimi Doost:** methodology, writing – review and editing. **Mohammad Hashem Hashempur:** conceptualization, methodology, validation, resources, writing – review and editing, project administration, supervision.

## Conflicts of Interest

The authors declare no conflicts of interest.

## Transparency Statement

The lead author, Mohammad Hashem Hashempur, affirms that this manuscript is an honest, accurate, and transparent account of the study being reported, that no important aspects of the study have been omitted and that any discrepancies from the study as planned (and, if relevant, registered) have been explained.

## Supporting information

Supporting information.

## Data Availability

The corresponding author had full access to all of the data in this study and takes complete responsibility for the integrity of the data and the accuracy of the data analysis. The data set supporting the conclusions of this article is available from the corresponding author upon reasonable request.
