# Supplementary material for: Evaluation of Knowledge, Attitude, and Practice of Iranian Medical Students Toward Complementary and Alternative Medicine: A Cross‐Sectional Survey
Source: Health Sci Rep. 2025 Mar 12;8(3):e70539. doi: 10.1002/hsr2.70539 (PMC11903502; doi:10.1002/hsr2.70539)
Supplement: Supplementary file 1 — Supporting information. [file HSR2-8-e70539-s001.pdf]

Questionnaire

Dear Student, this study has been designed to examine your knowledge, attitudes, and practices (KAP) in various areas of complementary and alternative medicine (CAM). Before participating, please note that your participation is voluntary. Rest assured that your privacy and dignity will be protected. Your information will remain confidential, and your personal responses will not be disclosed.

"Conventional medicine" refers to a framework of medicine that is generally taught in medical schools, and sometimes the related medications are referred to by the public as "chemical drugs." CAM encompasses a wide range of therapeutic and health systems that are currently not recognized as part of conventional medicine. Additionally, in many cases in this questionnaire, the term CAM physicians refers to doctors with a specialized doctorate in traditional medicine in our country.

Gender: Female ○ Male ○ **Marital Status**: Single ○ Married ○ **Age**: ---- years

Year of medical education: First year ○ Second year ○ Third year ○ Sixth year ○ Seventh year ○

	Strongly agree	Agree	Neither agree nor disagree	Disagree	Strongly disagree
1- CAM can be beneficial in preventing of certain diseases.					
2- CAM can be beneficial in the treatment of specific diseases.					
3- Incorporating training related to CAM into the curriculum for general practitioners can be advantageous.					
4. The influence of belief or suggestion in patients often causes CAM to seem more effective than it truly is.					
5- Doctors should be able to address patients' general inquiries regarding CAM.					
6- The choice between conventional medicine, CAM, and a combination of both should be determined by the patient's specific condition.					
7- Patients should consult a traditional medicine specialist upon the recommendation of their conventional medical providers.					
8- Referring some patients to CAM practitioners is essential.					
9- We should inquire about patients' history of using CAM.					
10- CAM practitioners usually dedicate more time to their patients.					
11- The holistic and comprehensive approach of CAM towards the human					

body can enhance public acceptance of its practices.					
12- The limitations of conventional medicine in treating certain diseases may prompt patients to explore CAM.					
13- The introduction of CAM as an academic discipline in universities can lead to the standardization of its practices.					
14- Are you willing to actively participate in learning and studying CAM?					
15- CAM is generally more aligned with the cultural, emotional, and spiritual needs of most patients than conventional medicine.					
16- Individuals from lower economic and social backgrounds are more receptive to CAM.					
17- The introduction of CAM as academic disciplines in universities may result in the misaccreditation of them.					
18- CAM often conflicts with mainstream medicine.					
19- In many instances, patients incur lower costs for CAM treatments compared to conventional medical practices.					
20- Generally, CAM treatments have fewer side effects compared to conventional medicine treatments.					
21- The credibility of CAM in today's medical landscape is often perceived to be lower than it truly is.					
22- CAM should be examined and discussed solely within historical and philosophical contexts.					
23- CAM has no role or application in practical fields such as research and treatment.					
24- Would you consider utilizing CAM methods in the treatment of your patients in the future?					
18- Please respond to the following items based on your experiences and those of your family.	**I used it.**	**My family used it.**			
**Traditional Persian medicine**					
**Acupuncture**					
**Homeopathy**					
**Cupping**					
**Leech Therapy**					
**Massage Therapy**					
**Chiropractic**					
**Yoga**					

Please indicate your level of familiarity with the following items by selecting the appropriate options.	**Very familiar**	**Approximately familiar**	**Intermediate familiarity**	**Approximately unfamiliar**	**Completely unfamiliar**
**Traditional Persian medicine**					
**Acupuncture**					
**Homeopathy**					
**Cupping**					
**Leech Therapy**					
**Massage Therapy**					
**Chiropractic**					
**Yoga**					
